# Enhanced HMAX model with feedforward feature learning for multiclass categorization

**DOI:** 10.3389/fncom.2015.00123

**Published:** 2015-10-07

**Authors:** Yinlin Li, Wei Wu, Bo Zhang, Fengfu Li

**Affiliations:** ^1^State Key Lab of Management and Control for Complex Systems, Institute of Automation, Chinese Academy of SciencesBeijing, China; ^2^Institute of Applied Mathematics, Academy of Mathematics and Systems Science, Chinese Academy of SciencesBeijing, China

**Keywords:** HMAX, biologically inspired, feedforward, saliency map, middle level patch learning, feature encoding, multiclass categorization

## Abstract

In recent years, the interdisciplinary research between neuroscience and computer vision has promoted the development in both fields. Many biologically inspired visual models are proposed, and among them, the Hierarchical Max-pooling model (HMAX) is a feedforward model mimicking the structures and functions of V1 to posterior inferotemporal (PIT) layer of the primate visual cortex, which could generate a series of position- and scale- invariant features. However, it could be improved with attention modulation and memory processing, which are two important properties of the primate visual cortex. Thus, in this paper, based on recent biological research on the primate visual cortex, we still mimic the first 100–150 ms of visual cognition to enhance the HMAX model, which mainly focuses on the unsupervised feedforward feature learning process. The main modifications are as follows: (1) To mimic the attention modulation mechanism of V1 layer, a bottom-up saliency map is computed in the S1 layer of the HMAX model, which can support the initial feature extraction for memory processing; (2) To mimic the learning, clustering and short-term memory to long-term memory conversion abilities of V2 and IT, an unsupervised iterative clustering method is used to learn clusters with multiscale middle level patches, which are taken as long-term memory; (3) Inspired by the multiple feature encoding mode of the primate visual cortex, information including color, orientation, and spatial position are encoded in different layers of the HMAX model progressively. By adding a softmax layer at the top of the model, multiclass categorization experiments can be conducted, and the results on Caltech101 show that the enhanced model with a smaller memory size exhibits higher accuracy than the original HMAX model, and could also achieve better accuracy than other unsupervised feature learning methods in multiclass categorization task.

## 1. Introduction

Image categorization is a critical issue in computer vision and neuroscience research. As the natural images have a lot of variations in lighting, scale, shape, position and occlusion, extracting intrinsic features, which are not only invariant within same class but also discriminative between different classes, is the principle of the algorithms for image categorization. And the mechanisms and structures of the visual cortex, which support the robust recognition, are also the key points of neuroscience for visual cognition research. Traditional computer vision algorithms are far from perfect due to the aforementioned variations, while the visual system of the primates shows good performance in daily life. Thus, mimicking the structures, mechanisms and functions of the primate visual cortex to design visual algorithms will highlight computer vision researches, help to get an insight of the visual cortex and further promote the interdisciplinary study of computer vision and neuroscience.

In the last decades, many kinds of features have been proposed to represent the natural images in the field of computer vision. On the one hand, many global image representation methods are proposed, such as the subspace analysis methods including Principal Components Analysis (PCA) (Turk and Pentland, 1991) and Fishers Linear Discriminant Analysis (LDA) (Belhumeur et al., 1997), which can achieve compact holistic encoding but cannot deal well with partial occlusion or strong view changes; On the other hand, many elaborated local feature representation methods are designed, such as SIFT (Lowe, 2004) and SUFT (Bay et al., 2008), which are scale-invariant and robust to moderate viewpoint variations.

Moreover, a middle level representation method—Bag of Words (BoW) (Sivic and Zisserman, 2003), has achieved good performance for image-level classification. It extracts a collection of unordered local patches of a test image, and maps them to discrete visual words learned by k-means vector quantization (VQ), and then obtains a histogram feature vector for classification. As the BoW model does not encode spatial information, it can be invariant to position and pose, but lose discrimination in some conditions. In Lazebnik et al. (2006), Spatial Pyramid Matching (SPM) kernel is introduced to BoW, in which spatial information are encoded in different scales and better performance is obtained in scene classification task.

When compared with primate visual cortex, a majority of the traditional methods could be called as *flat processing methods*, in which features are designed and processed by task-dependent learning algorithms (Krüger et al., 2013), but the primate visual cortex is organized in a hierarchical structure, and has good generality and robustness in a various of visual tasks.

Thus, it could be meaningful to mimic primate visual cortex to design hierarchical computer vision algorithms. In this interdisciplinary research field, the groundbreaking work is the Nobel Prize work of Hubel and Wiesel (1959, 1962). Based on biological experiments on cats striate cortex (V1), they described a circuit model with simple cells and complex cells, in which the complex cell has a similar response characteristic as the simple cell, but has a larger receptive field and a higher level tolerance to variations. After that, many biologically inspired computational models for visual cognition are proposed, including the Neocognitron (Fukushima, 1988), the saliency-based visual attention model (Itti et al., 1998) and the HMAX model (Riesenhuber and Poggio, 1999; Serre et al., 2007), etc. Among them, the HMAX model is a feedforwad hierarchical feature learning model for classification task. It tries to mimic the structures and functions of the ventral stream of the primate visual cortex in the first 100–150 ms of visual cognition, and includes four layers (S1, C1, S2, C2) corresponding to the V1 to PIT layers of the primate visual cortex. By alternating between convolution operation in S layers and max-pooling operation in C layers, the model finally generates a set of position- and scale- invariant features.

However, the HMAX model has its shortages. Firstly, a random patch/prototype sampling method in C1 layer is used. The representation and discrimination ability of these patches are not guaranteed, and it doesn't mimic the higher level learning ability of the visual cortex (Gross, 2008; López-Aranda et al., 2009). Secondly, the model is only designed for binary classification task. A high feature dimension will be generated for its application in multiclass categorization task, as patches need to be sampled in each object class respectively, which decreases its generalization ability and is different from the memory process of the visual cortex (Gross, 2008; Tyler et al., 2013).

In recent years, many researchers tried to modify the HMAX model to improve its performance or introduce more biological mechanisms into it. Mutch and Lowe (2006), Huang et al. (2011b) refined the model with sparsification, lateral inhibition and feedback based feature selection for image classification. While Mutch and Lowe (2006) achieved patch selection based on the weights of SVM classifier, and Huang et al. (2011b) used a boosting method to learn discriminative patch. Both of them didn't consider the possibility of learning patch in an unsupervised manner. Walther et al. (2002) merged the saliency-based attention model (Itti et al., 1998) with the HMAX model to modify the response characteristics of the S2 layer, while we will try to introduce attention modulation in an early layer S1 to support the patch learning in the next layer (C1). Thériault et al. (2013) extended the coding and pooling mechanisms of the HMAX model with more scale and spatial information for robust image classification, but it didn't achieve patch learning as the original HMAX model. In addition, other modifications of the HMAX model demonstrated good performance in face recognition (Liao et al., 2013; Qiao et al., 2014a,b), scene classification (Huang et al., 2011a), and handwritten digit recognition (Hamidi and Borji, 2010). The corresponding properties of the HMAX and the BoW model to the human visual cortex were also investigated by Ramakrishnan et al. (2015).

Meanwhile, Deep Neural Networks (DNN), such as the Convolutional Deep Belief Network (CDBN) (Lee et al., 2009) and the Convolutional Neural Networks (CNN) (Krizhevsky et al., 2012), are also organized in a hierarchical mode. Although their correspondences to the structures and mechanisms of the visual cortex are not quite clear, they have shown good performance in image categorization task. However, these models are difficult to train because very large training sets are required to avoid overfitting, and most of the CNN models with the best performance (Girshick et al., 2014; Schroff et al., 2015) are supervised models.

Thus, in this paper, based on related biological researches (see more details in Section 2), we mainly focus on the first 100–150 ms feedforward feature learning process of the primate visual cortex (Lamme and Roelfsema, 2000; Pascual-Leone and Walsh, 2001) to extend the original HMAX model in the following aspects:

1. **Attention Modulation:** To mimic the bottom-up attention modulation (Theeuwes, 2010; Zhang et al., 2012) and the response characteristics of neurons in V1 layer (Chatterjee and Callaway, 2003; Donk and van Zoest, 2008), a saliency map is computed by combing the orientation and Lab color-space information together in the S1 layer of the HMAX model based on the concept of local feature contrast. The generated salient regions mainly correspond to salient objects, and the boundary and resolution of objects are well kept. The proposed salient regions are taken as the initial candidate regions for feature extraction.
2. **Memory Processing:** To mimic the conversion of short-term memory to long-term memory of V2 (López-Aranda et al., 2009) and the learning, selectivity and clustering ability in distributed regions of inferotemporal cortex (IT) (Gross, 2008), a memory processing method with two steps is proposed to replace the random prototype sampling method in the HMAX model. Firstly, multiscale middle level patches are densely extracted in the salient regions. Secondly, the patches are selected with an unsupervised iterative clustering method. During which, rare and meaningless patches are deleted, and similar patches are grouped in the same cluster, and a classifier for each cluster is also learned. Thus, each cluster can be taken as a distributed region of IT layer, which contains neurons with similar selectivity of memory. Furthermore, the patches in each cluster mainly correspond to critical parts of objects, which are discriminative and representative. Due to the unsupervised learning mode, similar patches from different objects are shared, which would support the multiclass categorization task with less memory.
3. **Feature Encoding and Multiclass Categorization:** Corresponding to the distributed memory regions with similar discrimination ability (Gross, 2008), the Gaussian-like operation in S2 layer of the HMAX model is replaced by classification operation of each cluster. To mimic the feature encoding in Milner and Goodale (2008), the maximal activation of each cluster in the C2 layer of the HMAX model and its relative spatial position are cascaded as the final feature vector. Softmax is taken as the decision layer for multiclass categorization, and each output corresponds to the distributed associated regions of different objects for visual cognition in the cortex (Tyler et al., 2013).

The remaining parts of this paper are organized as follows. In Section 2, the related biological researches supporting the work of this paper are discussed. In Section 3, a brief introduction of the HMAX model is given, and the detailed improvements and methods of our work are proposed. In Section 4, multiclass categorization results on Caltech101 are given, and comparison experiments with other models are also discussed. Finally, in Section 5, we conclude this paper and discuss the results and our future work.

## 2. Related biological researches

As the HMAX model and its modifications in this paper try to mimic the structures and mechanisms of the ventral stream of primate visual cortex, the review of related biological researches in anatomy, neurobiology and cognitive science that support the whole HMAX framework and the modifications are discussed respectively as below.

### 2.1. Biological researches of the HMAX framework

The ventral stream of primate visual cortex is associated with complex shape discrimination, object recognition, attention and long-term memory (Merigan, 1996; De Weerd et al., 1999; Nassi and Callaway, 2009). It is organized in a hierarchical way, after getting its inputs from the lateral geniculate nucleus (LGN), the visual information goes through V1, V2, V4 to areas of IT: PIT, Central inferotemporal(CIT), and anterior inferotemporal (AIT) successively.

In the ventral stream, as receptive fields of neurons in one visual layer together represent the entire visual field, each layer contains a full representation of the visual space. During the processing, visual information is propagated from a local region to its succeeding hierarchical region, in which the receptive field size of a neuron is approximately 2.5 times larger than the input layer. Such convergent connectivity overlaps continuously with each other and ensures the invariant representation of visual stimuli. Please refer to Serre et al. (2007) for more detailed biological evidence of the HMAX model.

### 2.2. Biological researches of the modifications

#### 2.2.1. Neuronal response characteristic and feature encoding mode

The orientation, position and color information are critical for feature encoding in visual cognition.

##### 2.2.1.1. Orientation and location

The neuronal responses of V1 can discriminate small changes in visual orientations and spatial frequencies, and the spatial location of visual information is well retained. V2 and V4 are similar with V1, but have more tuning properties. The responses of V2 neurons could also be modulated by the orientation of illusory contours, and discriminate whether the stimulus is part of the foreground or the background (Qiu and von der Heydt, 2005). V4 is tuned for object features of intermediate complexity, like simple geometric shapes. IT layer is associated with the representation of complex object features.

##### 2.2.1.2. Color

The processing of color information begins in the retina with three types of cones cells-L, M, S, which have different responses to different wavelength lights (Hunt, 2005). Then the signals are transmitted through LGN to V1. The color cells in LGN and V1 are only sensitive along two axes, roughly red-cyan and blue-yellow (Wiesel and Hubel, 1966; Chatterjee and Callaway, 2003; Field et al., 2007). In V1, there are double-opponent neurons which compute local color contrast and color constancy (Danilova and Mollon, 2006; Kentridge et al., 2007). V1 color cells are clustered within cytochrome-oxidase blobs, and then project to the cytochrome-oxidase thin stripes of V2, which in turn project to globs in PIT. Glob cells achieve the perception of hue including red, green, blue, and to some extent yellow (Conklin, 1973). The final processing of color signals takes place in IT, which may help with shape decision making (Matsumora et al., 2008; Conway, 2009).

Finally, the visual inputs are transformed into representations that embody the enduring characteristics of objects and their spatial relationship (Milner and Goodale, 2008).

#### 2.2.2. Attention modulation

Attention modulation includes two modes: bottom-up and top-down. Visual selection is completely stimulus-driven in the first 150 ms, and the salience of objects can be modulated by bottom-up priming in a passive automatic way. In the later time (N150 ms), through massive recurrent feedback processing, active volitional control based on expectancy and task will bias visual selection in a top-down manner (Theeuwes, 2010).

In this paper, we focus on the bottom-up attention modulation, which is associated with salience. It is computed on the basis of the detection of locations which have significant local feature contrast, along some dimension or combination of dimensions (Itti and Koch, 2001; Donk and van Zoest, 2008). Firstly, a bottom-up saliency map can be created in V1 (Theeuwes, 2010; Zhang et al., 2012), and lateral connections (Gilbert and Wiesel, 1983; Rockland and Lund, 1983) between V1 neurons help mutual suppression between neurons tuned to similar input features. In addition, V2 is mainly responsive to top-down modulations (Beck and Kastner, 2005). In V4, bottom-up saliency and top-down control converge, and finally generate an overall saliency map (Töellner et al., 2011a,b).

#### 2.2.3. Distributed memory and association structure

The regions in the ventral stream have distributed memory and association structures.

Layer 6 of V2 are found to be important in the storage of object recognition memory and the conversion of short-term object memories into long-term object memories (López-Aranda et al., 2009). IT is connected with other memory associated areas, namely the hippocampus, the amygdala and the prefrontal cortex. Gross (2008) revealed that neurons in IT with similar selectivity of memory are clustered together and they also display learning ability over time. For example, different neural populations appear to be selectively tuned to particular components (e.g., face, eyes, hands, legs) of the same biological object.

Moreover, discrete object categories are even associated with different regions: objects with many shared features (typical of living things) are associated with activities in the lateral fusiform gyri, whereas objects with fewer shared features (typical of nonliving things) are associated with activities in the medial fusiform gyri. While Perirhinal cortex (PRC) in the anteromedial temporal lobe (aMTL) is associated with discrimination between highly similar objects (Tyler et al., 2013). In addition, the Parahippocampal Place Area (PPA) could differentiate between scenes and objects, and the Fusiform Face Area (FFA) is more sensitive to facial and body recognition rather than to objects (Spiridon et al., 2006).

## 3. Methods and detailed implementation

In this part, the HMAX model is firstly reviewed. Secondly, based on the biological researches stated above, our enhanced model, focusing on the first 100–150 ms unsupervised feedforward cognitive process of the primate visual cortex, is proposed. And the modifications and methods are discussed in details.

### 3.1. The HMAX model

During the hierarchical processing, the HMAX model progressively increases its selectivity and invariance for recognition. The function of each layer in the HMAX model is discussed briefly in the following.

#### 3.1.1. S1 layer

This layer mimics the simple cells in V1, which have a Gabor-like response characteristic. The grayscale input image is processed by a convolution operation with multidimensional array of S1 cells, and the S1 cells act with Gabor function as follows

(1) $$G(x,y)=\text{exp}(-\frac{x_{0}^{2}+\gamma^{2}y_{0}^{2}}{2\sigma^{2}})\times \text{cos}(\frac{2\pi }{\lambda }x_{0})$$

where *x*_0_ = *x*cosθ + *y*sinθ and *y*_0_ = − *x*sinθ + *y*cosθ. 4 orientations θ (0°, 45°, 90°, and 135°) and 16 scales *s* are selected, and other parameters are also tuned to generate 64 (= 4 × 16) S1 layer feature maps **FM_S1_**, see Table I in Serre et al. (2007), for more details.

#### 3.1.2. C1 layer

This layer mimics the complex cells in V1, which have larger receptive fields than simple cells in V1 (S1 layer) and show some degree of tolerance to shift and scale. Each C1 layer feature map is generated by max-pooling local neighborhoods (*L*_*S*_ × *L*_*S*_) in the same scale band with a step overlap, as Equation (2). Here, one scale band is formed by two feature maps with adjacent scales in S1 layer. Thus, some degree of shift and scale invariance is achieved in C1 layer, and 32 (= 4 × 8) C1 layer feature maps **FM_C1_** are obtained.

(2) $$FM_{C1}{\left(x,y\right)}^{s,\theta }=\underset{{}_{u_{x,y}\in B\_FM_{S1}{}^{s,\theta }}}{\max}u_{x,y}$$

where **u**_*x, y*_ are the local neighborhoods centered at point (*x, y*) in one of the orientation map of one scale band of S1 layer—**B_FM_S1_**^*s*,θ^.

#### 3.1.3. Prototype sampling

In this stage, *M* prototypes {*P*} are extracted from the C1 layer across all four orientations (*n* × *n* × 4), and *n* = (4, 8, 12, 16) is the prototype size. Only a random sampling method is used for prototype extracting. For binary classification task, the prototypes are only sampled from the positive training set.

#### 3.1.4. S2 layer

This layer corresponds to the cells in V4 and IT layer. For all positions and orientations of each scale band, the difference of the one feature map patch *X^s^* ∈ **FM_C1_**^*s*^ centered at (*x, y*) and each prototype *P*^*m*^ ∈ {*P*} is computed in a Gaussian-like way as Equation (3).

(3) $$FM_{S2}{\left(x,y\right)}_{m}^{s}=\text{exp}(-\beta \|X^{s}-P^{m}\|)$$

Where β defines the sharpness of the tuning. Here, as all the four orientations are computed together, 8 × *M* S2 layer feature maps **FM_S2_** are computed.

#### 3.1.5. C2 layer

In this layer, for the **FM_S2_** corresponding to one prototype *P*^*m*^, its C2 layer response is computed by taking a global maximum over all scales and positions. Thus, the final feature vector consists of *M* C2 values, which is a position- and scale- invariant representation of an image.

### 3.2. The enhanced HMAX model

**Figure 1:**
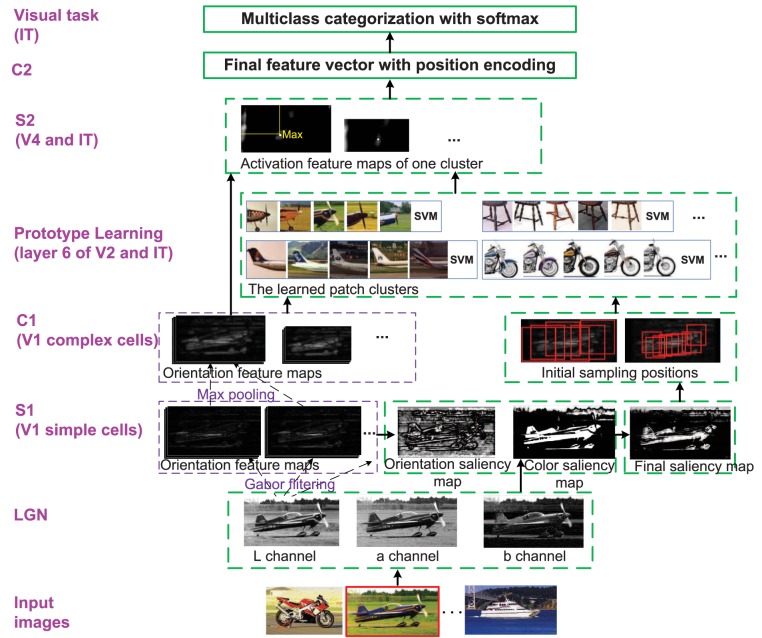
**The whole framework of the enhanced HMAX model**. Each layer corresponds to the region in visual cortex. Besides the functions and structures of the original HMAX model, the modifications are marked with dotted green bounding box. The image with red bounding box on the bottom is the current processing image. LGN is sensitive to Lab color space; S1 (simple cells in V1 layer): The bottom-up saliency map based on orientation and color contrast is computed; C1 (complex cells in V1 layer): The initial patches are sampled by centering on the selected salient points; Prototype learning (V2 and IT): An iterative clustering method is used to learn discriminative patch clusters and their classifiers, which corresponds to memory processing; S2 (V4 and IT): Each cluster classifier is used as a detector to generate S2 layer; C2: Final features are integrated with orientation, position (and color) information; Visual task (IT): Multiclass categorization with softmax are achieved.

Given a set of training images D and N, where D is a “discovery dataset” comprising a variety of object classes, and N is the“natural world dataset” including many other common objects and scenes. The goal of the enhanced HMAX model is to mimic the first 100–150 ms feedforward visual cognition procedure with the images in D and N by introducing attention modulation, memory processing and position encoding into the original HMAX model, and finally achieve multiclass categorization. The whole framework of this paper is given in Figure 1. All the modifications of the original HMAX model are discussed in the following, which correspond to related biological researches that stated in Section 2.

#### 3.2.1. Attention modulation—saliency map generation

In this step, the original HMAX model is extended with attention modulation in S1 level, in which a bottom-up saliency map is generated based on color and orientation contrast, which corresponds to the biological evidence of attention modulation in V1 layer (Gilbert and Wiesel, 1983; Donk and van Zoest, 2008; Theeuwes, 2010). Only the dataset D is processed in this step, as it contains the object class to be learned. The generated saliency map will support the prototype learning in next stage.

Different from the gray input images in the original HMAX model, we use color input images and convert them to Lab images, as this color space is mostly consistent with the characters of LGN and V1 cells, which are sensitive along two axes, roughly red-cyan and blue-yellow (Danilova and Mollon, 2006; Kentridge et al., 2007).

For a color image, based on the work of Itti et al. (1998) and Achanta et al. (2009), firstly, the S1 layer orientation feature map with 12 orientations θ and 16 Gabor scales *s* are computed based on the L channel in Lab color space. Since all the feature maps have the same size of the original image, we can directly compute the orientation saliency map by difference operation as Equation (4) rather than the downsampling and interpolation operation in Itti et al. (1998). Here, the first 8 scales are selected to compute the orientation saliency map. The scale interval Δ*s* for the difference operation is 4, and the difference of all the scales and orientations are added together to get **SFM_O_**. Then, by computing the mean value *avg*() and the standard deviation *std*() of **SFM_O_**, the normalized orientation saliency map **SFM_O_** is obtained.

(4) $$\begin{array}{l} SFM_{O}=\sum_{s\;=\;1}^{4}\sum_{\theta \;=\;1}^{12}\left(FM_{O}{}^{s,\theta }-FM_{O}{}^{s+\Delta s,\theta }\right)\\ \bar{SFM_{O}}=(SFM_{O}-avg(SFM_{O}))/std(SFM_{O}) \end{array}$$

Secondly, the Lab color feature map **FM_C_** is obtained by gaussian filtering of the original Lab image, and the color saliency map **SFM_C_** is computed as Equation (5). $avg(FM_{C}^{i})$ computes the mean value of the *ith* channel of **FM_C_**, and the normalized color saliency map **SFM_C_** is computed in the same way as **SFM_O_**.

(5) $$SFM_{C}=\sum_{i\;=\;l,a,b}(FM_{C}{}^{i}-avg(FM_{C}{}^{i}){)}^{T}(FM_{C}{}^{i}-avg(FM_{C}{}^{i}))$$

Where *l, a, b* corresponds to the three channels of Lab color space, respectively.

Finally, the normalized saliency feature maps of color and orientation are combined together as **SFM** = λ_1_ · **SFM_O_** + λ_2_ · **SFM_C_** to get the final saliency map (λ_1_ = 0.4, λ_2_ = 0.6). The procedure of saliency map generation is illustrated in the S1 layer of Figure 1. Furthermore, the salient points are also sorted according to their values in **SFM**.

#### 3.2.2. Memory processing—prototype learning

The prototype selection of the original HMAX model (Serre et al., 2007) is based on random sampling. The representation and discrimination ability of these prototypes are not guaranteed. While in other modified HMAX models (Mutch and Lowe, 2006; Huang et al., 2011b), prototypes are selected or learned in each object class, respectively in a one vs. all manner, which is a supervised procedure.

However, we try to mimic the first 100–150 ms in visual cognition, which is an unsupervised feedforward procedure. Thus, we modify the unsupervised middle level patch (prototype) discovery method in Singh et al. (2012) to adapt to the HMAX framework. In the new model, patches belonging to multiclass can be learned without image label in an iterative way. During this procedure, similar patches are clustered together and one classifier is learned for each cluster for discrimination. This procedure corresponds to the memory processing function of V2 and IT, as the layer 6 of V2 are found important for the conversion of short-term memories to long-term memories (López-Aranda et al., 2009), and neurons in IT with similar selectivity of memory are clustered together and they also display learning ability over time (Gross, 2008).

In the new model, the datasets D and N are divided into two equal, non-overlapping subsets (*D*_1_, *N*_1_ and *D*_2_, *N*_2_) for cross-validation. The unsupervised prototype learning can be achieved in two phases: *initial sampling* and *iterative learning*. The iterative learning is alternately processed between two steps: clustering and training classifiers on the two subsets. In addition, multi-scale patches are extracted, and the patches with different size *n*(=16, 28) are processed independently in the prototype learning procedure, and finally integrated together in the C2 layer.

In the initial sampling phase, the patches from *N*_1_ are taken as negative samples and selected in a random sampling manner with an overlap constraint, which filtrates the randomly sampled centers by making the distance between the any two centers no smaller than $\frac{1}{4}$ of the patch size *n*. The patches from *D*_1_ are sampled in the salient regions. We discuss the initial sampling method in *D*_1_ in the following.

Firstly, 8 C1 layer feature maps **FM3_C1_** are computed with Equation (2). As the patches are sampled in the first scale band of C1 layer **FM_C1_**^1^, the corresponding positions of the sorted salient points in C1 layer are computed. The final salient points are selected sequentially with an overlap constraint, which is the same as the constraint of the random sampling method on *N*_1_. Then, *S* middle level patches {*P^D^*} in **FM_C1_**^1^ are extracted by taking the final selected salient points as centers, which could guarantee a good cover of the whole salient region as well as avoid big overlap between patches.

**Figure 2:**
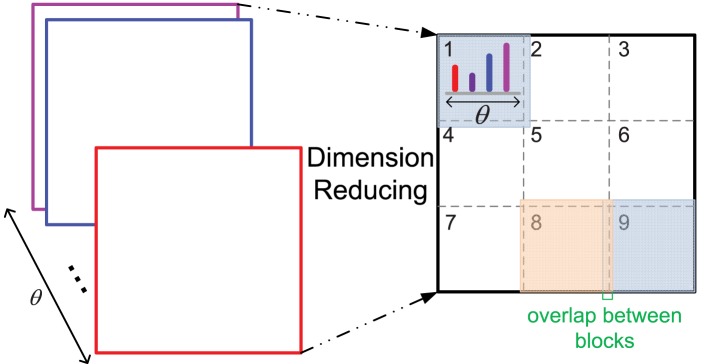
**The dimension reducing method**. As 12 orientations θ are used, the original feature dimension of a patch with size *n* is 12 × *n*×*n*, *n* = 16, 28 in this paper. After dimension reducing processing, the feature dimension is 12 × 9 for all the patches with different size *n*.

Furthermore, due to the bigger size of middle level patches and more orientations computed than those of the original HMAX model, the feature dimension of a patch is high, which could be difficult for the SVM training of each cluster in the iterative learning step, as there are very little positive training data. Thus, a dimension reducing method is proposed, which is similar to the design of HoG features (Dalal and Triggs, 2005) (illustrated in Figure 2). One patch is divided into 3 × 3 blocks with an overlap, and the orientation histogram of each block is computed, normalized with L2 norm, and cascaded to form the final feature vector of a patch, which is an effective and concise representation of a patch. In some cases, since the IT layer is sensitive to the RGB color space (Conklin, 1973), the RGB color histogram can also be computed in the same way of orientation histogram (dividing into 2 × 2 blocks), and added to the final feature vector.

In the iterative learning phase, the initial sampled patches are further learned and clustered.

Since the traditional k-means clustering method is not fruitful for the middle level patches due to its low level distance metric, in order to learn discriminative patches and avoid overfitting, an iterative learning method is used.

Secondly, by taking the patches of a cluster as positive features and all randomly sampled patches {*P*^*N*^} in *N*_1_ as negative features, a weighted linear SVM classifier is learned for each cluster. And the SVM classifier is used as a detector in the first C1 scale band of *N*_1_ to find hard negative patches, which are then used to retrain the SVM classifier of each cluster. Then, the learned SVM classifier of each cluster is used as detector in *D*_2_, and only the top *q* (= 5) ranked patches are taken to update the corresponding cluster to keep the purity. If the top ranked patches are less than 3, the cluster is deleted. Then, the subsets *D*_1_, *N*_1_ and *D*_2_, *N*_2_ are switched and a new iteration with SVM training and cluster updating are processed. In experiments, the algorithm converges in 4–5 iterations.

Moreover, the purity and discriminativeness of each learned cluster *K*_*i*_ is computed as Equation (6).

(6) $$\begin{array}{l} purity(K_{i})=\frac{1}{r}\sum_{j\;=\;1}^{r}Score_{SVM}(P_{j}),\;\;P_{j}\in K_{i}\\ discri(K_{i})=FireNum_{D}/(FireNum_{D}+FireNum_{N}) \end{array}$$

Where *Score*_*SVM*_(*P*_*j*_) is the score of the *jth* patches in the *ith* cluster *K*_*i*_ computed with the corresponding SVM classifier, and *r* is set to 10 (*r* > *q*) to evaluate the generalization of the cluster. *FireNum*_*D*_ and *FireNum*_*N*_ are the firing rates of the SVM classifier of cluster *K*_*i*_ in the datasets D and N, respectively.

The purity and discriminativeness are normalized in the same way as Equation (4), and the general score of each cluster is computed with the normalized purity and discriminativeness, defined as *score*(*K_i_*) = *purity*(*K_i_*) + λ_3_ · *discri*(*K_i_*). Finally, the top ranked clusters and their corresponding classifiers are represented as $\Omega^{n}\;=\;{\left\{K_{i},C_{i}\right\}}_{i=1}^{\Gamma_{n}}$ (Γ_*n*_ is the number of patches with size *n* = 16, 28), and all the clusters with different size *n* are stored together as $\Omega \;=\;{\left\{K_{i},C_{i}\right\}}_{i=1}^{\Gamma },\Gamma =\Gamma_{16}+\Gamma_{28}$.

The whole prototype learning algorithm is given in Algorithm 1.

**Algorithm 1:** Unsupervised Prototype Learning Algorithm

**Input:** Training set T including D and N
**Output:** The top ranked clusters and their corresponding clusters $\Omega \;=\;{\left\{K_{i},C_{i}\right\}}_{i=1}^{\Gamma }$
1: D ⇒ {*D*_1_, *D*_2_}; N ⇒ {*N*_1_, *N*_2_} ⊳ Split D and N into equal sized disjoint subsets
2: Compute **FM_C1_** with Equation (2) ⊳ Compute C1 layer feature maps
3: **for** one patch size *n* in {16, 28} **do**
4: Select *S* points from the sorted salient points ⊳ Operate in the first scale band of **FM_C1_** of *D*_1_
5: Extract *S* patches {*P^D^*} with dimension reduction
6: ${\left\{K_{i}\right\}}_{i\;=\;1}^{S/5}⇐Kmeans(\{P^{\text{D}}\})$ ⊳ Use Kmeans to divide patches to *S*/5 clusters
7: **while** not converged **do**
8: **for** all *i* that *size*(*K*_*i*_)≥3 **do** ⊳ Maintain clusters with enough patches
9: *C*_*i*_ ⇐ *SVM_train*(*K*_*i*_, *N*_1_) ⊳ Use weighted SVM to train classifier for each cluster
10: *Hard_N*_1_ ⇐ *hard_mine*(*C*_*i*_, *N*_1_) ⊳ Find the hard negative patches in *N*_1_
11: $C_{i}^{new}⇐\;\text{SVM\_retrain}\left(K_{i},Hard\text{\_}N_{1}\right)$ ⊳ Retrain the classifier with *Hard_N*_1_
12: $K_{i}^{new}⇐\;detect\text{\_}top\left(C_{i}^{new},D_{2},q\right)$ ⊳ Find top *q* = 5 patches in *D*_2_
13: **end for**
14: *K*⇐*K*^*new*^; *C*⇐*C*^*new*^
15: *swap*(*D*_1_, *D*_2_); *swap*(*N*_1_, *N*_2_)
16: **end while**
17: compute $score\left(K_{i}\right)=\bar{purity\left(K_{i}\right)}+\lambda_{3}\cdot \bar{discri\left(K_{i}\right)}$ based on Equation (6)
18: $\Omega^{n}\;=\;{\left\{K_{i},C_{i}\right\}}_{i=1}^{\Gamma_{n}}⇐select\text{\_}top\left(C,score,\Gamma_{n}\right)$ ⊳ Select the top Γ_*n*_ clusters of each patch size
19: **end for**
20: Unite all the top ranked cluster Ω^*n*^ with different patch size *n* to $\Omega \;=\;{\left\{K_{i},C_{i}\right\}}_{i=1}^{\Gamma },\Gamma =\Gamma_{16}+\Gamma_{28}$

#### 3.2.3. Feature integration with position encoding

In this part, the final feature vector in C2 layer with orientation and spatial position is computed.

Firstly, for each cluster {*K*_*i*_, *C*_*i*_} in Ω, its corresponding S2 layer feature maps are generated by using *C*_*i*_ as detector in all the scale bands of the C1 layer. Each unit in the S2 layer is a SVM score, which could intuitively represent the discrimination ability of the *ith* cluster that corresponds to a distributed memory region of object component in IT (Gross, 2008). Finally, 8 × Γ S2 layer feature maps are obtained.

Then, the C2 layer features are computed in the same way of the original HMAX. But the relative position coordinate (*x*_*max*_/*W, y*_*max*_/*L*) of the maximum score of each cluster classifier is also added to the final feature vector, and *W, L* are the width and length of the S2 layer feature map with the maximum score in it. Thus, the length of the C2 layer feature vector of an image is 3 × Γ. Here, by integrating appearance features and loose spatial constraint together, more representative and discriminative features are learned, which is consistent with the function of the ventral visual stream (Milner and Goodale, 2008).

#### 3.2.4. MultiClass categorization

Based on the unsupervisedly learned features in C2 layer together with the image labels, a softmax layer is added on the top of the C2 layer to achieve the multiclass categorization task. Each output of the softmax layer corresponds to a distributed association region of an object class (Tyler et al., 2013). In addition, due to the unsupervised iterative learning manner of $\Omega \;=\;{\left\{K_{i},C_{i}\right\}}_{i=1}^{\Gamma }$, similar patches from same object class are gathered together, and in some conditions, similar patches from different object class are also clustered together. The features from multiclass are shared, and the memory storage could be small. Meanwhile, the discriminativeness and purity are also guaranteed. Thus, the final feature vector is compact and suitable for multiclass categorization task.

## 4. Results

Multiclass categorization experiments on Caltech101 are carried out. The implementation of each modification and the final categorization result of the proposed model are evaluated and discussed. Furthermore, the comparison experiments with the original HMAX model and other unsupervised feature learning methods on multiclass categorization are also conducted and analyzed.

### 4.1. Dataset

Caltech101 (Fei-Fei et al., 2007) is a dataset with 102 classes (101 object class and 1 background). Here, 10 object classes are selected, and 30 color images are randomly sampled in each class to form the “discovery dataset” D (positive training set). The 437 color images in the background class are taken as the “natural world dataset” N (negative training set). During the testing process, another 20 color images in each of the 10 object classes are selected to form the testing set.

### 4.2. Saliency map generation and salient point selection

**Figure 3:**
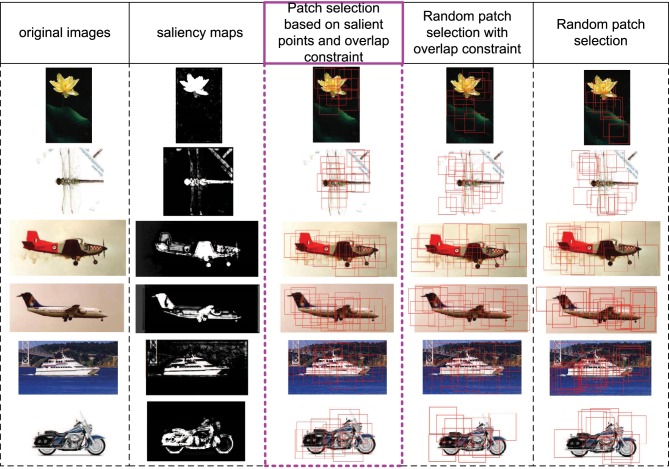
**Some image examples, their saliency maps, and the initially sampled patches (red bounding boxes) with different methods**. The 1st column includes original images, the 2nd column includes saliency maps computed based on Equations (4) and (5). The 3rd column includes initially sampled patches extracted by taking the final selected salient points as centers, which is used in this paper. The 4th column includes randomly sampled patches but with the overlap constraint (same with the constraint of 3rd column). The 5th column includes purely random sampled patches.

In this part, we discuss the role of saliency map in S1 layer (corresponding to V1 layer). Firstly, the V1 layer does have the ability of bottom-up saliency map generation based on local contrast. Secondly, the saliency map in S1 layer could provide a good initial region for patch selection. In Figure 3, some images, their corresponding saliency maps, and initially selected patches with different methods are given. We can see that the generated salient regions of our saliency map computation method (column 2) correspond to object regions in images, and the boundary and content are well kept. The proposed initial patch sampling method based on salient points (column 3) has a dense cover of the whole object region as well as avoid big overlap between patches, while the random sampling method with only overlap constraint (column 4) has a wider cover of the whole image, which extracts some meaningless patches in the background. Moreover, the purely random sampling method (column 5) has extracted some highly overlap patches, which is redundant, and can not guarantee a good cover of the whole object region.

**Figure 4:**
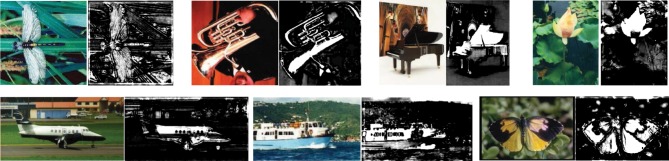
**Images with complicated backgrounds (left) and their saliency maps (right)**. Although some points in the complicated backgrounds are activated, the dominant object regions still have more salient and continuous activations.

For images with more complicated backgrounds, some saliency maps generated by the proposed method are also given in Figure 4. Although some points in the backgrounds are also activated, the object regions still have more salient and continuous activations.

### 4.3. Memory processing—prototype learning

**Figure 5:**
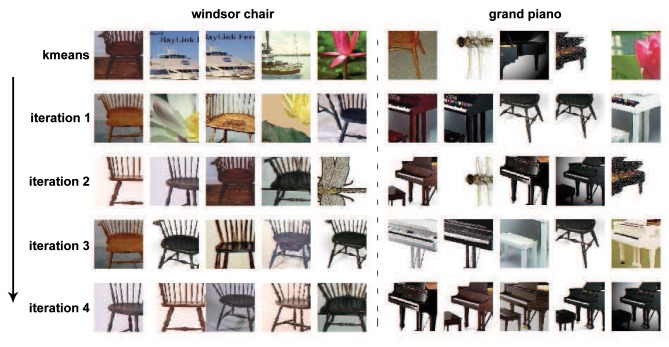
**The iterative learning procedure of two clusters**. The initial clustering with k-means is not quite meaningful because the patches in one cluster don't belong to same or similar part of objects. With the unsupervised iterative prototype learning method, the patches in one cluster become more and more similar. After 4th iterations, the patches in one cluster correspond to same critical part of objects.

By processing the initially sampled patches with the unsupervised iterative patch clustering method in **Algorithm** 1, similar middle level patches are clustered together, and their corresponding SVM classifiers are also obtained. The convergence procedure of two clusters is given in Figure 5. Before the first iteration, the cluster is generated by k-means clustering, and there are some noises because of the low level distance metric. After 4 iterations, the middle level patches that clustered together become more similar.

**Figure 6:**
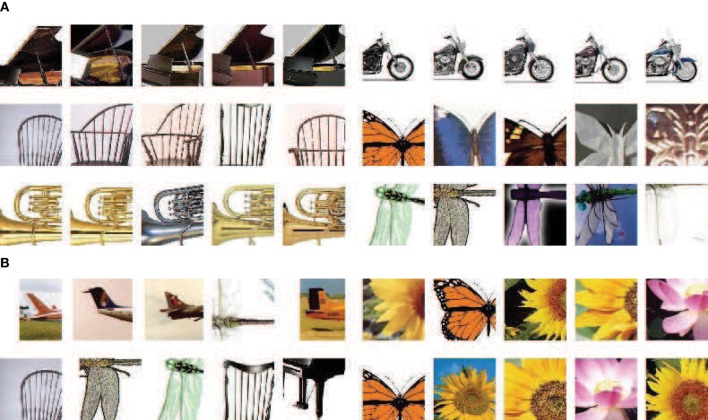
**Some examples of the final learned clusters**. In **(A)** (6 clusters), for each cluster, its patches correspond to same critical part of objects, which indicates the prototype learning method has learned representative features; In **(B)** (4 clusters), similar patches from different objects are clustered together, which shares the memories of different object class and helps to save memory size.

Some examples of the final learned clusters are given in Figure 6. For each cluster in Figure 6A, the middle level patches correspond to a kind of key parts of an object class, which are representative and discriminative. While in Figure 6B, although the patches in same cluster are from different object classes, their appearances in orientation feature space are similar, which indicates that the similar middle level patches from different object class could be shared. Finally, by combining the middle level patches and the corresponding SVM classifier together, each cluster could be taken as a distributed region selective to one kind of object parts in the IT layer of the visual cortex.

### 4.4. Categorization results and comparisons

In this section, the multiclass categorization results of the enhanced HMAX model (eHMAX) are discussed in a various of conditions and compared with the original HMAX model (oHMAX). In addition, because the features of the eHMAX are learned in an unsupervised way, and each learned cluster could be considered as a true visual word (see Figure 6), and in the C2 layer the relative position coordinate of each cluster is also encoded into the final features, we could see that the framework of the eHMAX is similar with the BOW and SPM framework. Thus, the comparison experiments of the eHMAX and the representative models with BOW and SPM framework are also conducted, which includes KSPM (Lazebnik et al., 2006), ScSPM (Yang et al., 2009), and LLC (Wang et al., 2010).

**Figure 7:**
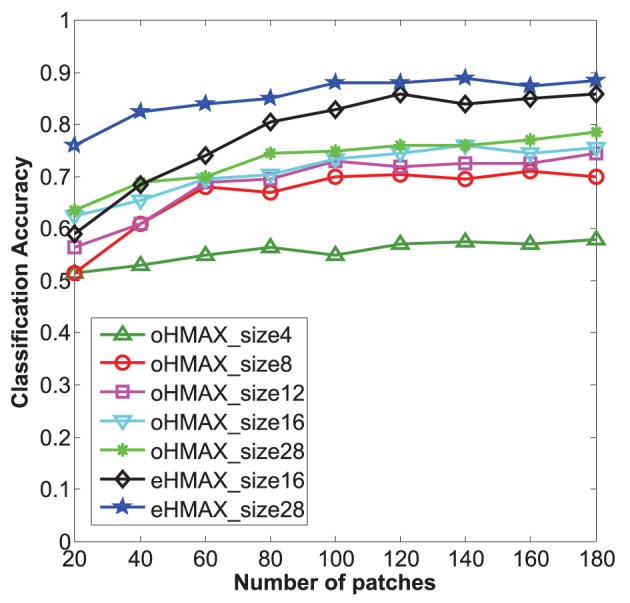
**Categorization accuracy of 10 classes in Caltech101 with different methods**. The size number of each line corresponds to the used patch size of each model. The bigger the patch size, the higher accuracy can be achieved for all the models. The eHMAX with patch size 28 has the highest accuracy in all the conditions, which indicates that the memory storage and feature representation of the eHMAX model is more compact and effective.

Firstly, the categorization results of the eHMAX and the oHMAX with different sizes and different numbers of patches are given in Figure 7. Here, the number of patches in the eHMAX corresponds to the number of clusters, as each cluster generates one feature map in the S2 layer, which is same with function of one patch (prototype) in the oHMAX.

As shown in Figure 7, with same number of patches, the patches with bigger size have shown higher accuracy in both models. It is because that the patch size 28 is much closer to the middle level patches, which always correspond to critical parts of object. While the patch size *n* = 4, 8 is too small to contain enough discriminative information. Moreover, the eHMAX model has shown better accuracy than the oHMAX model almost in all the conditions. For example, when the number of patches is 100, the accuracy of the eHMAX with patch size 16 and 28 is 83 and 88%, respectively, which is 9.5 and 13% higher than the oHMAX with 100 patches sized at 16 and 28. This indicates that the learned clusters in the eHMAX are more discriminative and representative. In order to achieve higher accuracy, more number of patches is needed for the oHMAX. And in some conditions, the increase of number of patches can not improve the accuracy a lot because of the low discrimination ability of randomly sampled patches. For example, the accuracy of the oHMAX model with 1000 patches sized at 16 and 28 is 80.5 and 81.5%, respectively. The improvements are not that dramatic comparing with the performance with the configuration of 100 patches. In a word, the memory storage and feature representation of the eHMAX model is more compact and effective.

In addition, We find that without encoding the relative spatial position information, the accuracy of the eHMAX model with patch size *n* = 16, 28 (100 clusters) drops to 79 and 83.5%, respectively. It is obvious that besides the learned discriminative and representative clusters, the good performance of the eHMAX model is also partly dependent on position encoding.

Secondly, according to the numbers of selected top clusters in different patch size, the final results of the eHMAX by combing mutiscale clusters are given in Table 1, and the results of other models are also listed. In the eHMAX Model, by combining 100 clusters sized at 28 and 500 clusters sized at 16, the best performance is obtained as 92.5%, while the oHMAX model with same number and scale of patches has an accuracy of 83%. For the oHMAX in Serre et al. (2007) with 4 patch sizes [4,8,12,16] and 800 patches of each size, the accuracy is only 78.5%. In addition, by setting the dictionary size of KSPM, LLC and ScSPM model to 600, which equals to the number of clusters in the eHMAX model, the ScSPM model achieves the best performance as 91%, but the accuracies of these three models are still lower than the eHMAX.

**Table 1:** **Categorization accuracy of 10 classes in Caltech101 with different models**.

**Model**	**Parameters**	**Accuracy (%)**
eHMAX	Patch size: [16,28], Number of clusters: [500,100]	**92.5**
oHMAX	Patch size: [16,28], Number of patches: [500,100]	83
oHMAX	Patch size: [4,8,12,16], Number of patches: [800,800,800,800]	78.5
KSPM	Dictionary size: 600	85
ScSPM	Dictionary size: 600	91
LLC	Dictionary size: 600	89.5

*The best accuracy is achieved by the eHMAX model as 92.5%, and it is bold to be more striking*.

## 5. Discussion

Different from the original HMAX model with a random patch/prototype sampling method, and other modified HMAX models with selection of patches in a supervised manner, we focus on the first 100-150 ms feedforward/unsupervised cognitive processing to enhance the HMAX model, its success mainly depends on attention modulation, memory processing and feature encoding abilities, which are designed based on the related biological researches.

In the experiments, it is clear that the attention modulation could generate saliency maps with high quality, and provide good candidate salient regions/points for patch learning. The memory processing procedure could learn discriminative and representative middle level patches in an unsupervised iterative manner. Meaningless patches are deleted and similar patches from same/different object classes can be gathered in a same cluster during the procedure, which indicates the memory selectivity, sharing and clustering ability of the enhanced HMAX model.

As for the multiclass categorization experiments on Caltech101, the performance of the enhanced HMAX model and the original HMAX model with different size and number of patches is evaluated. Both of the models could achieve higher categorization accuracies with bigger size of patches, which indicates the middle level patches (*n* = 28) contain more discriminative information. The categorization accuracies of the two models have no significant improvement when the number of the patches is bigger than 100. For the enhanced HMAX model, the reason may be that the purity and discrimination of the new clusters are lower than that of the first 100 clusters. For the original HMAX model, the reason may be the new randomly sampled patches are meaningless or redundant. Furthermore, the enhanced HMAX always has a better performance than the original HMAX model with the same size and number of patches, with the reason that the enhanced HMAX model learns more discriminative middle level patches and also encodes relative position information into features. All in all, the enhanced HMAX model can achieved higher performance with smaller memory storage.

In addition, the comparison experiments of the HMAX model and three representative BOW and SPM models are conducted, which include KSPM, ScSPM, and LLC model. These three models also learn features in an unsupervised way, and their dictionary/codebook is similar to the patch cluster in the enhanced HMAX model. But the visual words in the KSPM and the ScSPM models are SIFT descriptors with patch size *n* = 16, and the visual words in LLC model are HOG descriptors with three sizes, *n* = 16, 25, 31, respectively. They are all extracted from the original image level, and these three models are flat processing method.

The experiment results indicate that the enhanced HMAX model has a higher accuracy than the above three models, which may owe to its hierarchical modeling and the discriminative middle level patches. Firstly, the hierarchical modeling helps to achieve some kind of invariance. Secondly, the size of the middle level patches is *n* = 16, 28 in the C1 layer (C1 layer is five times smaller than the original image), and the middle level patches mainly correspond to critical parts of objects, which are much bigger than the SIFT and HOG descriptors.

## 6. Conclusion

In this paper, based on recent biological research findings, we modified the original HMAX model by mimicking the first 100–150 ms unsupervised feedforward visual cognition process. The main contributions include:

1. A bottom-up saliency map is generated based on local orientation and color contrast in S1 layer, which mimics the attention modulation ability of V1 layer of the visual cortex. The boundary and content of salient object are well kept, and the points in the salient regions are selected to support the initial sampling of patches.
2. An unsupervised iterative clustering method is used to learn more representative and discriminative middle level patches, which mimics the learning, clustering and short-term memory to long-term memory conversion abilities of V2 and IT layer. After a few iterations, the patches in each cluster almost correspond to the same or similar key parts of object class, and one classifier of each cluster is also learned to distinguish it from others.
3. The feature vector is computed in C2 layer, which is the cascade of the maximum activation value of each cluster and their corresponding relative spatial position. Finally, a softmax decision layer is used to achieve the multiclass categorization. This process mimics the feature encoding mode and distributed associated regions of different objects in the visual cortex.

Experiments on multiclass categorization task have demonstrated the effectiveness of the enhanced HMAX model.

In the future, on the one hand, we will investigate the reinforcement learning ability and the recurrent feedback processing of the visual cortex, and mimic the related structures and mechanisms to build new biologically inspired visual models. With the labels of images, the saliency map generation and memory learning can be further reinforced in a supervised manner, and a higher accuracy and robustness could be expected. With the ground-truth bounding box of objects, the relative position of each patch to the center of each object could be encoded to support categorization as well as detection task. On the other hand, it will also be meaningful to find a way to achieve multiple visual tasks, such as classification, detection and segmentation, in an unsupervised or weakly supervised way, since this way requires less human labor and the primate visual cortex does have such ability.

## Author contributions

YL prepared the methods of attention modulation, memory processing and position encoding. WW provided the related biological researches, which inspired the design of the whole framework. YL and FL conducted the experiments. YL, WW, and BZ prepared the manuscript. BZ initiated this study and supervised all aspects of the work. All authors discussed the results and commented on the manuscript.

### Conflict of interest statement

The authors declare that the research was conducted in the absence of any commercial or financial relationships that could be construed as a potential conflict of interest.
